# Draft Genome Assembly of the Entomopathogenic Bacterium Photorhabdus luminescens subsp. sonorensis Caborca

**DOI:** 10.1128/MRA.00692-19

**Published:** 2019-09-05

**Authors:** Duy An Duong, Patricia Espinosa-Artiles, Rousel A. Orozco, István Molnár, S. Patricia Stock

**Affiliations:** a Center for Insect Science, University of Arizona, Tucson, Arizona, USA; b Southwest Center for Natural Products Research, University of Arizona, Tucson, Arizona, USA; c Department of Entomology, University of Arizona, Tucson, Arizona, USA; d School of Animal and Comparative Biomedical Sciences, University of Arizona, Tucson, Arizona, USA; University of Maryland School of Medicine

## Abstract

Photorhabdus luminescens subsp. sonorensis strain Caborca is an entomopathogenic bacterium with a dual lifestyle, namely, as a mutualist of the Heterorhabditis sonorensis nematode and a pathogen to a wide range of insect species. The genome assembly, in 231 contigs, is 5.2 Mbp long and includes 25 putative gene clusters for secondary metabolism.

## ANNOUNCEMENT

Photorhabdus luminescens subsp. sonorensis strain Caborca is the symbiont of the entomopathogenic nematode Heterorhabditis sonorensis, a native species from Caborca, Sonora State, Mexico (1). P. luminescens subsp. sonorensis and *H. sonorensis* form an insecticidal partnership that targets a wide range of insect hosts (2). Upon death, the insect cadaver serves as a suitable environment that enables the nematodes to mature and reproduce. P. luminescens subsp. sonorensis produces a variety of toxins and secondary metabolites with diverse antimicrobial properties. Mass spectrometry analysis of crude extracts of P. luminescens subsp. sonorensis cultures revealed 15 distinct compounds (3), 8 of which are unique compared with those of other *Photorhabdus* spp. These findings prompted further efforts to investigate this bacterium for potential applications in agriculture and medicine.

Two rounds of genome sequencing using the Ion Torrent next-generation sequencing technology were conducted. In the first round, genomic DNA (gDNA) was isolated from overnight cultures grown in Luria-Bertani medium inoculated from a primary phase colony, using the Thermo Scientific genomic DNA purification kit for bacterial gDNA isolation. The recovered gDNA was resuspended in 100 μl nuclease-free water and sequenced with Ion Torrent technology with a 200-bp chemistry on a 314 chip at the University of Arizona Genetics Core (UAGC). In the second round, gDNA was extracted from an identical culture using phenol/chloroform/isoamyl alcohol (25:24:1), followed by ethanol precipitation, and sequenced at the Pennsylvania Genomic Analysis Core (University of Pennsylvania) with Ion Torrent technology on an Ion S5 system with a 540 chip.

Raw sequencing data were checked for quality prior to trimming to ensure high-quality data for the assembly process using Sickle 1.0 (4) (quality threshold, 20; min length, 50) and Trimmomatic-programmable 0.36 (5) (LEADING, 25; TRAILING, 25; MINLEN, 50) launched from the University of Arizona Cyverse Discovery Environment (https://de.cyverse.org/de/). The quality of data was analyzed using FastQC 0.11.5 (https://www.bioinformatics.babraham.ac.uk/projects/fastqc/), also launched from the Cyverse Discovery Environment. *De novo* assembly was completed using the Geneious 11.1.5 software (https://www.geneious.com) with the plugin assembler SPAdes 3.10.0 (6) with different k-mer sizes to select the best assembly. The quality of the assemblies was assessed using QUAST (7). Table 1 displays a summary of the best assembly from each data set.

**TABLE 1:** Summary of the assemblies from different data sets

Feature	Value for data set(s):		
	First	Second	Combined
No. of raw reads	395,197	7,801,769	8,196,966
No. of trimmed reads	314,277	6,288,858	6,603,135
No. of contigs	509	263	231
Length of contigs (bp)	135–70,803	202–262,598	202–431,332
G+C content (%)	42.4	42.5	42.5
Avg coverage (×)	9.4	252.2	272.0
Total length (bp)	5,145,620	5,216,436	5,225,282
*N*_50_ (bp)	19,121	70,534	88,967
*L*_50_	82	19	16

The assembled genome from the combined data set was annotated with the NCBI Prokaryotic Genome Annotation Pipeline (8), resulting in the identification of a total of 4,657 genes, including 4,132 protein-coding sequences, 109 RNA-coding genes, 416 pseudogenes, and 7 to 13 partial rRNA sets. antiSMASH (9) analysis predicted 25 gene clusters that are potentially involved in secondary metabolism, including that for the canonical compound isopropylstilbene, which is produced by many *Photorhabdus* spp. (10), as well as those for novel compounds that await to be identified and tested for their antimicrobial properties.

### Data availability.

The raw reads have been deposited in the NCBI SRA under the accession no. SRS4934682. This whole-genome shotgun project has been deposited at DDBJ/ENA/GenBank under the accession no. SBIJ00000000. The version described in this paper is the first version, SBIJ01000000.
